# Product Quality Assessment in the Internet of Things: A Consumer-Oriented Approach

**DOI:** 10.3390/s22062215

**Published:** 2022-03-12

**Authors:** Mohammed Sharief Abdelrahman Naem, Mouloud Koudil, Zineeddine Ouldimam

**Affiliations:** 1 Department of Computer Science, Rabak Technical College, Sudan Technological University, Khartoum 11111, Sudan; 2 LMCS, Ecole Nationale Supérieure d’Informatique, Oued Smar 16309, Algeria; m_koudil@esi.dz (M.K.); fz_ouldimam@esi.dz (Z.O.)

**Keywords:** IoT, product-quality, assessment, a consumer-oriented-approach, IoT recommender

## Abstract

This paper proposes a consumer-oriented approach to IoT product recommendations. It is designed to help new consumers choose high-quality IoT products that best meet their needs. We used hybrid techniques to implement the proposed approach. Experiments were also conducted to implement an intelligent IoT marketing system at the Rehab marketplace. The system has shown good results in its performance, usability, and user satisfaction. These results confirm the applicability and effectiveness of the approach in assessing and recommending IoT products.

## 1. Introduction

In 1999, Kevin Ashton devised the term “Internet of Things” at Procter & Gamble (P&G). He discussed his vision of a new computer application that collects data around us to automatically control many daily activities [1].

The Internet of Things (IoT) has become a rapidly growing model for smart applications, relying on the rapid development of wireless communications, and advanced sensing/actuation technologies [2,3].An IoT product represents any smart solution developed based on IoT technology, including devices, service, application appliances, platform, and ecosystems [4]. It integrates various software/hardware components, where IoT devices are connected together over the Internet to collect data and then exchange and analyze information [5]. These products are usually designed to address real-world problems according to a specific application scenario, such as wearable devices, advanced metering infrastructure, agricultural drones, autonomous robots, remote monitoring systems, fleet management, fire alarm systems, etc. [1,6,7].

Nowadays, IoT markets are growing at a rapid rate; service providers have provided a wide range of IoT-based solutions to facilitate various aspects of life. Huge numbers of IoT devices were produced and countless services were created. For instance, Libelium and Telus IoT markets have more than 300 solutions covering various sectors [8,9]. Due to the market pressure and the increasing demand for IoT products, IoT markets are filled with many poor-quality products especially in terms of device quality [10]. It should be noted that a wide range of low-cost IoT devices are being produced everywhere, while many of them are being released without sufficient testing. This can greatly affect quality assurance and customer satisfaction. Furthermore, some functions may be ignored or reduced, making these devices vulnerable to multiple limitations and threats [11,12,13,14].

These technical issues greatly affect the ability of potential IoT consumers to get IoT products that meet their desires and requirements. Many of them want to take advantage of smart products to facilitate some aspects of their daily life. However, they do not have enough experience to get quality products. Often, they do not find any useful recommendations on which products are best for their needs [4,15,16]. 

To tackle this situation, the paper proposes a consumer-oriented approach to help new consumers choose IoT products that best serve their needs from products available in the market. Based on the quality definitions, satisfying a consumer’s requirements on a particular product indicates that it is a high-quality product. Hence, the proposed approach aims to satisfy these requirements by filtering the lists of announced IoT products using machine learning techniques. It mainly focuses on assessing the features of IoT products based on the experiences of past consumers and quality experts. Then, it recommends to new consumers the best quality products. The hybrid approach can be an effective machine learning approach to do the job. It allows combining content-based filtering (CB) and collaborative filtering (CF) techniques, to obtain accurate predictions as well as heuristics. As a result, a set of useful recommendations will be generated to help new consumers, allowing them to make the right decisions before buying or using any product and saving their money.

To validate the proposed approach, a case study was presented for an IoT marketplace called “Rehab”. An Intelligent IoT Marketing System (IIoTMS) was developed to help a large group of consumers. The study found that the new system effectively contributed to serving them and achieved satisfactory results. The rest of the paper is organized as follows: Section 2 is the related work. Section 3 introduces the methodology. Section 4 provides experimental setups. Section 5 presents the results. Section 6 provides a discussion. Section 7 provides a conclusion.

## 2. Related Works

This section reviews related work, as two main aspects associated with this article will be covered: (1) Quality assessment of the IoT. (2) IoT based recommendations. 

Regarding the first aspect, many studies indicate that the IoT model still lacks a unified interpretation of quality. It does not yet have a quality reference model that can be used to assess the quality of its products. Therefore, several studies have attempted to suggest relevant models inspired by conventional software quality models, as in [17,18,19,20]. Despite these efforts, the quality characteristics associated with conventional software are not well suited to the IoT. It has unique features that distinguish it from conventional software, such as mobility, automation, interoperability, scalability, etc.

In the same vein, some studies have sought to develop a quality model that fits the requirements of IoT. In [21,22], the authors provided a catalogue of non-functional requirements to eliminate conflicts between the quality characteristics in HCI ubiquitous computing and IoT applications. It is a general catalogue that can be used to deal with various aspects during the development of IoT applications. In [23], the authors provided an estimation approach to assess the quality of IoT applications. This is an approach based on a multi-dimensional matrix of QoS factors. Nevertheless, we also noticed that researchers do not agree on the factors that influence the quality of IoT.

Regarding the second aspect, the development of recommendation systems for IoT is a new arena that is attracting a number of researchers [24]. There are endeavors to implement IoTSRS in a variety of IoT domains but they face many challenges compared to traditional recommender systems. In [25], the authors proposed a personalized recommendation system. They have developed a multi-objective recommendation model to solve the big data overload caused by internet vehicles, abundant environmental and mobile data. The study was used a real-world dataset and focused on examining recommendation accuracy, recommendation novelty, and coverage. In [26], the authors proposed a framework for evaluating the performance of the Recommendation interface. The framework allows users to adjust their own characteristics and goals. Further, a deep neural network is trained to predict the efficiency of recommendations. In [27], the authors proposed an approach to assess trust and reputation of things in a multi-internet of things (MIoT) scenario. Among these scenarios, they discussed MIoT architecture of a large shopping center consisting of several buildings. Every building has a smart device that is connected to other devices in other buildings, including temperature sensors, video surveillance, fire sensors, presence sensors, etc. The customer’s personal device can act as a personal shopper providing them with appropriate suggestions (RS). This is actualized by interacting with other MIoT objects, browsing through other stores’ offerings, and then using machine learning algorithms to improve the quality of recommendations. In this proposal, the authors focus on the concept of trust and reputation of IoT devices. From a literature review, the closest work to this study is discussed in [28]. It is an IoT service recommendation (IoTSRS) that has been proposed to recommend users based on the objects they own. The researchers suggested a hyper-graph based model for the IoT recommendation system.

In [29], the authors extended the previous proposed model, while also studying the usefulness of adopting the traditional recommendation approaches on IoT service recommendation. In [30], the authors proposed an IoT food recommender inside a refrigerator for healthy living. This is a system to recommend food recipes to the user with the ability to identify the raw food available in the refrigerator and ensure their safety.

## 3. Methodology

This section introduces the research methodology. It discusses several considerations that will be taken into account in assessing the quality of IoT products. It also explains a set of processes needed to formulate useful recommendations that can help new consumers.

### 3.1. Quality Assessment Considerations

Quality has been defined by ISO in (8402—1986) standard as: “The totality of features and characteristics of a product or service that bear on its ability to satisfy stated or implied needs” (ITU-T 2008). In another update of the (900—2005) standard, ISO definition was developed to become “The degree to which a set of inherent characteristics fulfil requirements” [31,32]. 

Based on these definitions, the study assumes that we can objectively decide whether the product is good or not, and even useful or not, according to the user’s experiences (past consumers, quality experts). Past consumers can identify and assess the quality features of IoT products they have used before. They can also rate similar products and identify new features based on their impressions. Quality experts can also help identify/verify features of new products as well as rate each product.

### 3.2. Systemic Processes 

In general, consumer needs can be classified into two main types: (1) Explicit requirements. (2) Implicit requirements [31]. Satisfying both explicit/implicit requirements on products means that they are of sufficient quality from a consumer perspective. However, this requires a goal-oriented process to exclude low quality products. 

**Figure 1:**
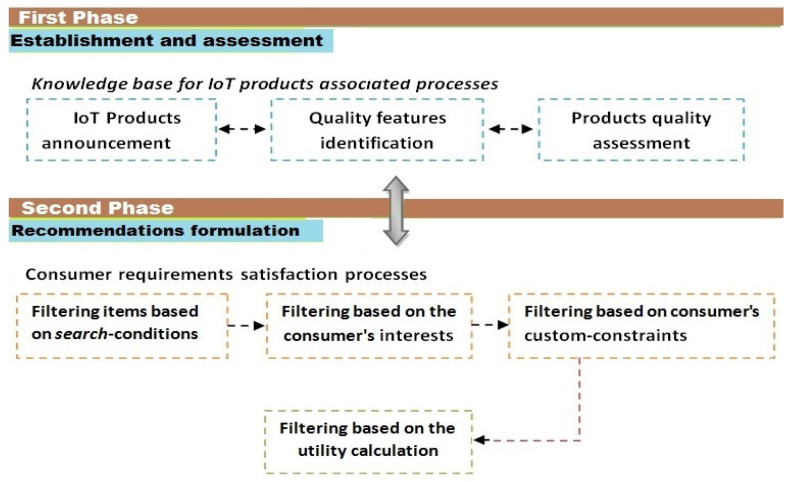
A methodology for assessing and recommending IoT products.

The proposed methodology defines two main phases for achieving this goal: (1) Establishment and assessment. (2) Recommendations formulation. Each phase includes several processes, which will be implemented as potential function of a hybrid recommendation system. There are different actors that must interact to perform the related activities according to predefined roles and permissions, including IoT suppliers, quality experts, and past consumers. To describe the sequence of phases and their associated processes, we present a hierarchical diagram, as shown in Figure 1.

The recommendation system (RS) is among the most popular and influential Artificial Intelligent applications around the world [33], which can be defined as “software tools and techniques providing suggestions for items to be used by a user” [34]. “Item” refers to the type of specific recommendation given to users, such as for products, movies, books, etc. RS is a collection of opinions, evaluations, impressions, or even knowledge given by some people in the form of initial recommendations (input), and then aggregated for subsequent use by others (appropriate recipients) [25,26,33,34]. 

The most important requirement for building an RS database is to identify the key objects of the system (items, users) and the associated relationships [33,34]. It is necessary to classify the data accurately to facilitate the inference of useful information (i.e., consumer preferences or user ratings). In the same context, there are two main concepts that play an important role in evaluating the quality of recommendations in this type of application: (1) Trust. (2) Reputation. Trust is a concept based on personal and subjective events. These events are described by factors and evidence. Reputation is a collective measure of trust, and these measures are based on impressions or ratings from members of the community [27].

#### 3.2.1. Establishment and Assessment Phase

**Figure 2:**
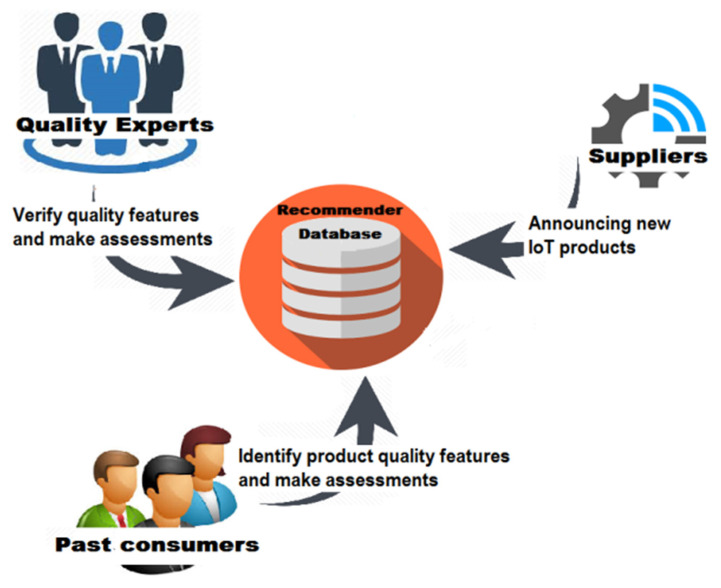
The establishment and assessment phase.

This phase is necessary to *create a knowledge base for IoT products available in the market*. It defines a set of activities needed to collect information about these products, as shown in Figure 2. These activities will be carried out by RS users using three main processes, including product announcement, quality features identification, and product assessment.

**Figure 3:**
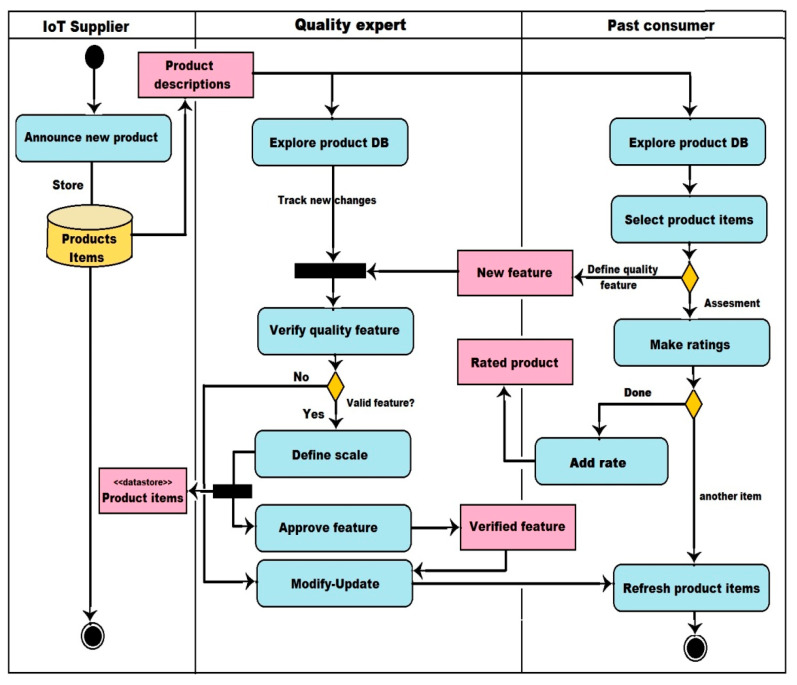
The main actors’ related activities.

In this scenario, each person connected to the RS is interested in a particular type of activity that is somehow related to shopping. Users play many roles ranging from vendors, suppliers, or consumers, to quality experts. It is a framework similar to a MIoT architecture, where users are located in many places and use different types of smart devices, including smartphones, laptops, smart glasses, etc. To represent the interaction of actors, a UML activity diagram was designed to illustrate the sequence of activities, as shown in Figure 3.

The supplier (IoT vendors) represents a group of IoT-based organizations that offer a variety of smart IoT solutions, such as service providers, device vendors, etc. Quality expert represents a group of people who have enough experience in IoT products. The experts will be involved in various assessment-related tasks such as verifying the quality features of IoT products, determining appropriate metrics and weights, and rating new products. Past consumer represents a group of people who have previously used IoT products (past users). They have their own impressions of various IoT products. Thus, they will play an important role in enriching the rating process by assessing the quality of the newly announced products based on their experiences (rating features, defining new features). 

##### Products Announcement

Smart IoT products are often delivered in several forms, such as services, applications, systems, platforms, etc., with different use cases [35,36,37]. 

This process is dedicated to gathering necessary information about recently released IoT products in the markets. IoT suppliers are supposed to periodically announce their released products in a central database. Products information will be shared between various actors to assess these products. 

##### Quality Features Identification

Quality features are mentioned in the literature using several synonyms such as attributes, qualities, characteristics, parameters, factors, or even aspects [38], depending on the context. For instance, any IoT product can have a number of quality features such as usability, portability, performance, etc. 

This process is concerned with assigning appropriate quality features to each declared product. It can also be described as an ongoing process in which related activities do not stop (machine learning process). Both experts and past consumers are responsible for determining the quality features of each new product based on their experience/expertise. However, the users who will play this role must have good awareness and sufficient experience. As a result, new features will be identified or assigned every time a new product is announced either manually by consumers/experts or automatically using some heuristics methods.

##### Products Assessment

**Figure 4:**
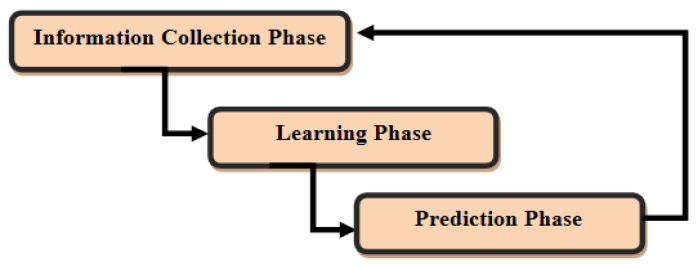
The learning-based assessment process.

This process is at the heart of our approach, which will be implemented as a machine learning process. A large amount of information can be used to predict consumers’ preferences scheme, including user-profiles and associated behaviors, as shown in Figure 4. 

Based on the assumption in Section 3.1, it focuses on subjective-assessment of the quality of IoT products based on the experiences of past consumers and quality experts (rating activities). The ratings of different users can be obtained by making a number of comparisons between the RS objects (products, users). 

Every IoT product can be assessed using both explicit and implicit ratings. The explicit rating is a direct way to obtain user opinions about a specific product or service. User ratings will be collected from past user preferences, including likes, star ratings, numerical ratings, etc. The implicit rating is an indirect way to obtain user opinions about a specific product or service. User ratings will be predicted by tracking users’ past behaviors, including clicks, views, favorites, and purchase data [33,35,39].

As a result, there are also various tools that can be considered for collecting user requirements directly or indirectly, including different requirements guidance such as conversational dialogs, profiles, etc. These tools are important for improving the recommender’s self-learning processes, by ensuring that enough information is collected from each consumer profile and multiple sources. In addition, they increase the RS’s ability to model consumer’s preferential behaviors (profiling).

#### 3.2.2. Recommendation Formulation Phase

This phase is used to formulate a set of useful recommendations to help new consumers using different filtering processes. Filtering is a term commonly used to describe the process by which certain elements are selected and others excluded according to predefined criteria or rules [16,40].

**Figure 5:**
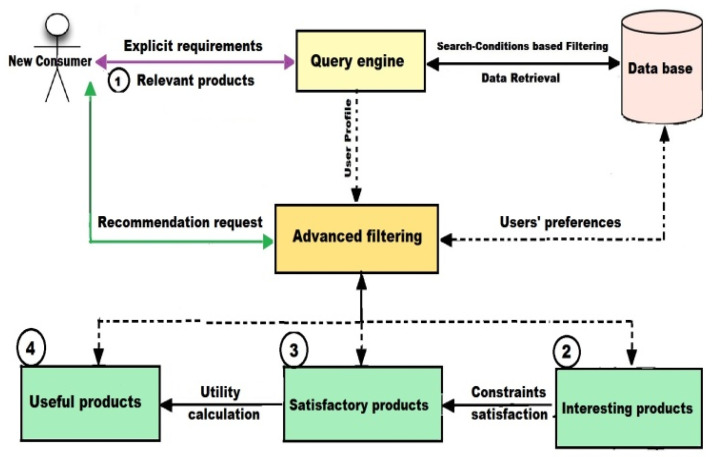
The recommendations formulation.

These recommendations are formulated by filtering IoT products to meet consumer implicit/explicit requirements, as shown in Figure 5. Explicit requirements represent direct user needs, which are keywords or conditions that a user places when searching for a specific product within the database. Implicit requirements represent a set of indirect user needs, such as interests, desires, custom constraints.

To satisfy the explicit consumer requirements, the user will be recommended with products declared in the databases that match their search keywords using a simple inquiry process, while satisfying the implicit requirements of the consumer requires advanced filtering processes using hybrid filtering rules. 

As a result, the new consumer will be allowed to obtain the highest quality IoT products available in the markets. They can also make the right decisions before purchasing any product using the utility function. The utility calculation can help the new consumer to understand the usefulness of each recommended product according to their needs.

##### Filtering Items Based on Initial Search-Conditions

This process is aimed to meet explicit consumer needs, representing the first level of recommendations that will be formulated. Each consumer should specify keywords for the IoT product(s) they want to purchase using a query engine. It is assumed that RS will respond by performing a query operation using given search conditions. Therefore, an initial list of products matching the search conditions will be generated by retrieving data from the database. 

Nevertheless, this process will produce low-quality recommendations, because the implicit requirements of the consumer are not yet recommended. The effectiveness of any RS depends on the validity of its recommendations in terms of users’ needs [33,39]. As a result, to efficiently respond to a user’s needs, the new consumer must request advanced recommendations to satisfy their implied requirements (interests, custom-constraints). Further filtering of the resulting lists using new conditions is required.

##### Filter Based on Consumer’s Interests

This process aims to reduce the number of IoT products resulting from the inquiry process by excluding products that do not suit the consumer’s preferences. A new shortlist of interesting products will be generated based on past consumers preferences, including the known past users’ ratings and other direct/indirect information.

**Figure 6:**
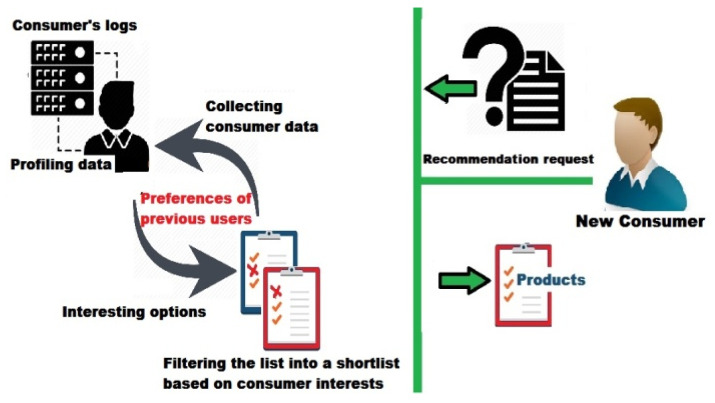
Filtering based on consumer’s interests.

The proposed RS must also be able to deep mine the past consumers’ log files to predict such interest-based recommendations, as shown in Figure 6. A mining process will be implemented to extract multi-attributes content for products that users have preferred in the past; whether they are items or contents.

The hybrid approach can be an effective machine learning approach in doing this task. It allows combining both of the collaborative and content-based filtering methods, to obtain accurate predictions as well as to generate heuristics [28,29,34,35,39,40].

There are two filtering rules that are necessary to make predictions during this filtering process: (1) Items similarity. (2) User similarity. These rules will be used to make various comparisons between the main RS objects (users, items) to predict interesting consumer products. However, these comparisons also require measuring the similarity between objects.

**Figure 7:**
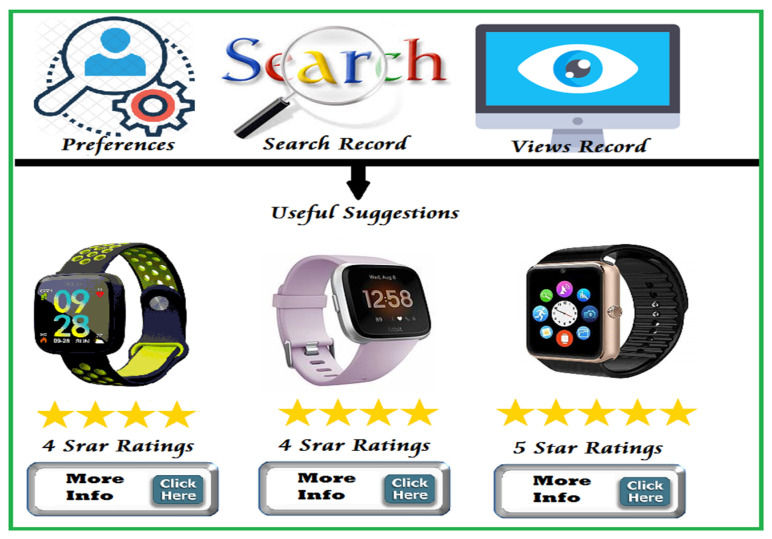
Example for recommending a user using items similarities.

Item similarity is a rule that can be used to provide suggestions to a user based on his previous choices [41]. This rule assumes that many useful recommendations can be inferred by using similar choices (Item-Item Filtering). A user can like the same items based on information derived from their search records, preferences, comments, views, etc, as shown in Figure 7. 

Item similarity can be applied in different ways to assist consumers, for example to suggest initial search conditions, or new product options with high-rates. It is a very effective rule when adopting a heuristic approach to predict new short-lists of IoT products that respond to consumer interests.

**Figure 8:**
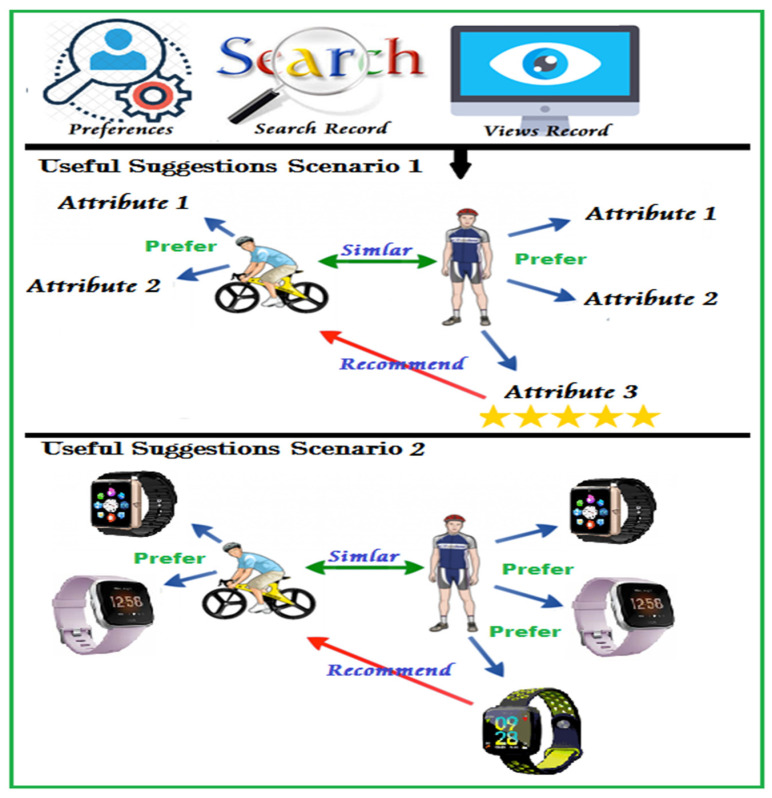
Example for recommending a user based on user similarities.

Users Similarities is a rule that can be used to check the differences between users by checking their similarities [33,42]. Sometimes, users may have the same preferences on a particular item or content, which may lead to infer that they have the same mood or interest, as shown in Figure 8. 

This figure illustrates two scenarios for user similarities, where the user–user filtering allows recommending items or contents based on user preferences. 

On the other hand, the term “similarity measure” refers to how similar data objects are in the recommendations system. It is commonly used in the context of data mining to measure the distance between objects in the dimensions that represent the features [43,44]. For instance, two objects become very similar when the distance between them becomes small, and vice versa. The similarity between any data object is measured within specific ranges. For example, the similarity of distance measures usually varies between [0, 1], as follows [45]:

(1) Similarity =1 if X = Y Where X, Y are two objects

(2) Similarity =0 if X ≠ Y

Although data objects may be different; points, vectors, or combinations, most of the similarity measures are subjective and applicable in many applications [33]. 

##### Filter Based on Constraints Satisfaction

Previous shortlist of interesting products may not meet the consumer’s personal constraints. This occurs because the consumer mood may not match the recommended options, or they may have custom constraints (personal interests). For instance, they may look for low prices, special brands or producers, specific quality features, etc. Thus, this process aims to satisfy the custom constraints of the new consumer, which seems to be a Constraint Satisfaction Problem (CSPs).

**Figure 9:**
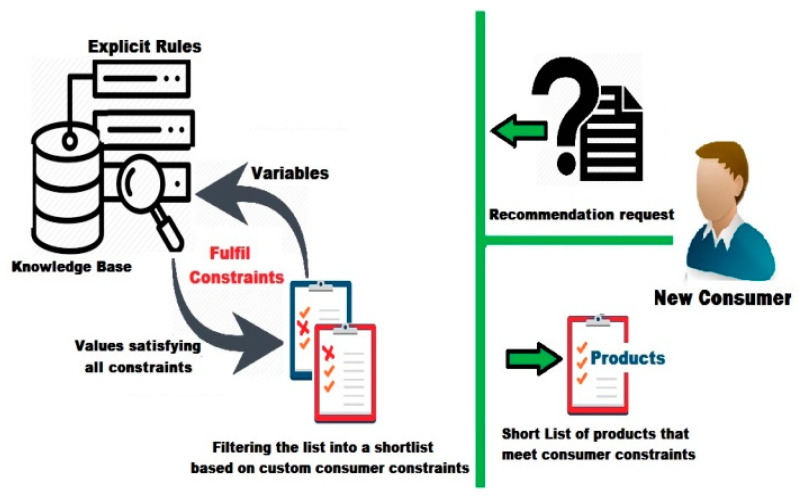
Filtering based on consumer’s constraints.

A solution for a CSP requires a concrete reproduction of variables (a set of values), where all specified constraints can be met [46]. In other words, it is the ability to assign a value to each variable in a way that satisfies all the constraints with these values [47]. In the same vein, creating a knowledge base about consumer requirements is essential to satisfy different types of constraints, as shown in Figure 9. 

A knowledge base can be used to define explicit rules. These rules are necessary to ensure that possible consumer constraints are managed using a finite set of variables, values, and constraints. It also allows linking user requirements to enable filtering of the previous shortlist. 

The special values related to the new consumer will be compared to the database entries, to filter the previous shortlist of products of interest. As a result, a new recommendation will be generated, which is a shortlist based on consumer constraints. It is another shortlist that is much closer to the real needs of a consumer. However, the consumer may need additional help to make the right decision by calculating the utility of the recommended options.

##### Filtering Based on the Item’s Utility

A useful recommendation can be defined as “a phase in which the calculated prediction is used to support the user’s decision by some recommendation processes” [34]. 

Filtering based on the item’s utility is a process used to calculate the utility of the recommended products to a new consumer. Although the previous shortlists are intended to satisfy the consumer’s needs (interests and constraints). However, some of the recommended options may not be very useful to the consumer. This occurs because filtering processes were considered only one dimension; single-criterion ratings (numeric or binary ratings of products by past users). 

We have considered adopting multi-criteria ratings to improve the quality of recommendations as well as better understand each user’s preferences. A single overall rating may hide the underlying heterogeneity of users’ preferences for different aspects of a given item. As a result, this RS should enable users to represent more complex preferences, by allowing them to specify their individual preferences and rate each item. The ratings provided will be used to formulate new recommendations based on multi-criteria ratings.

The utility function can be calculated according to the following formulas [34,44,48]:

(3) R: Users×Items→R0×R1×⋯×Rk

It is worth noting that the recommendations system is part of the information filtering systems. There are two main types of RS: (1) non-personalized. (2) Personalized. Non-personalized RS is a system that recommends to a user the most popular items, such as the most frequently purchased products, top-selling books, and top-downloaded movies. Personalized RS is a system that analyzes different data to formulate customized recommendations, including users’ profiles, users’ relationships, users’ purchases, etc.

Traditionally, RSs are based on methods like the nearest neighbor, clustering, and matrix factorization. Over time, these systems have also benefited from deep learning techniques. Deep learning has achieved tremendous success across multiple domains, including image recognition, natural language processing, context information, etc. There are many recent RS powered by complex deep learning systems, such as those at YouTube and Amazon [43,49]. These techniques can be generally considered to be sub-field of machine learning, which focus on learning the deep representations of data [26,50].

## 4. Experiments

This section presents the experimental setup used to implement the proposed approach. It aims to check the applicability of the approach and the accuracy of its recommendations.

### 4.1. Case Study Description 

Rehab is an offline marketplace with a physical location in downtown Khartoum. It sells different types of consumer electronics such as computers, phones, headphones, and accessories. It is also an IoT marketplace that contains a central platform for IoT solutions, including physical hardware, specialized software, and applications. 

The size of Rehab gradually increases, while users take a long time to purchase everything they need. For someone who is interested in working on an IoT project but is not sure where to start, they are expected to face many challenges to obtain a quality product that best suits their needs. It would be ideal if we had a smart application to help large groups of consumers undertake a simple shopping experience, especially the disabled, sick, and elderly.

As a result, experiments were conducted to develop an Intelligent IoT Marketing System (IIoTMS) at the marketplace. In addition, implementing a hybrid recommendation system as part of a centralized platform for IoT products can help solve these problems.

### 4.2. Application Scenario

As mentioned earlier, the proposed approach requires the participation of different users who may reside in different locations. This scenario requires developing a cloud-based system to allow each user to interact easily; most users prefer to use their smartphones. 

A number of smart technologies can be integrated into the IIoTMS environment for an easy shopping experience. It is assumed that once a new consumer reaches the marketplace, they will use the available smart glass. This glass will be used to run the shopping app and connect the consumer’s smartphone to the Internet. Indoor navigation (IN) technology has also been adopted to guide consumers in the marketplace. IN is one of the best navigation solutions [51]. It allows consumers to take the best and shortest route to check-out and place all required items in their shopping cart along the way.

As a result, each consumer will be able to browse the contents of the marketplace via a digital menu displayed on the smart glass, and check the availability of any of the product items with access to the specified location.

#### 4.2.1. System Architecture

**Figure 10:**
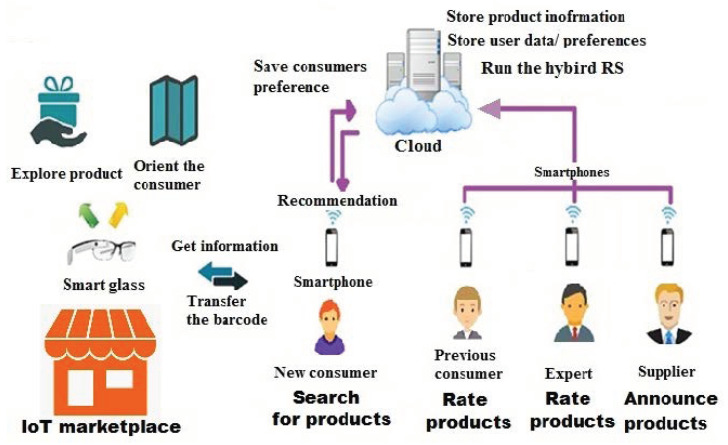
High-level system architecture.

To allow the modelling of required system functions, a high-level architecture for the IIoTMS has been provided. This architecture helps divide the functional areas of the system into higher level components, as shown in Figure 10. 

IIoTMS architecture consists of three layers, including cloud computing, smartphones, and smart glasses. Each layer provides a specific type of service, but each service may require one or more components to interact with one another. 

The cloud-based layer is a layer that used to store users and products data. It is also used to run the hybrid RS algorithm for consumers’ recommendation. The provided service will connect to smartphone applications via the Internet using the HTTP protocol to send and receive data. Smartphone-based layer is a layer designed to run a client-side recommendation service (medium-sized applications). This layer allows users connect to the Internet/other devices. It provides a user application that allows users to collaborate in assessing product quality. In addition, it will be connected to smart glass via WIFI or Bluetooth, to receive product barcodes and send information about each product. Smart glass-based layer is a layer used to store product locations in the marketplace. It represents a limited resource embedded system that is widely used to facilitate our life with many different useful features. Thus, the provided service will help new consumers search/access product items easily.

#### 4.2.2. System Deployment

**Figure 11:**
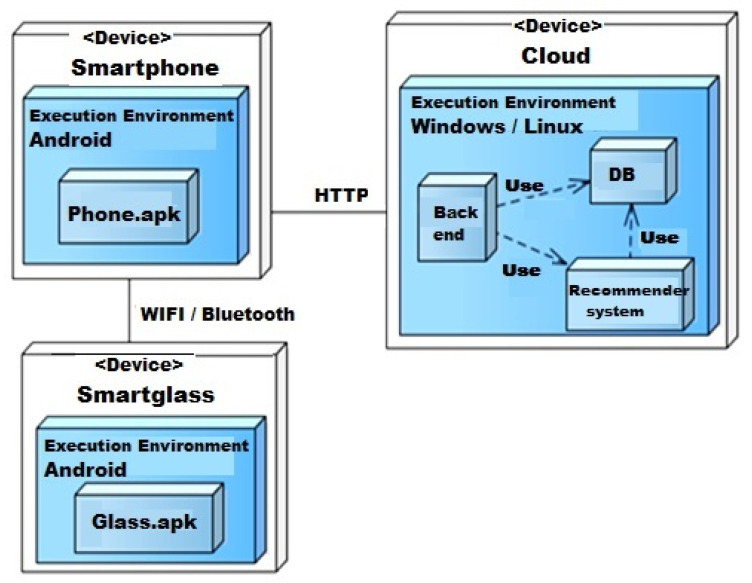
IIoTMS deployment diagram.

A deployment diagram has been created to show physical resources (hardware configuration), as shown in Figure 11. It focused on the actual runtime units and associations between them, including components, modules, nodes, connections, or any hardware/software required to complete the system. 

There are different types of devices that can be used to deploy the IIoTMS, including the cloud, smartphones, and smart glasses. Each device has an execution environment for implementing a specific set of components listed. It also requires many tools to implement system parts, including Android Studio, Node.js platform, Python, MongoDB, and IoT Dataset. System parts interact with each other as an integrated entity using different types of protocols, including WIFI, Bluetooth, and HTTP.

## 5. Results

This section presents the main findings of the study. The focus will be on measuring aspects related to IIoTMS implementation, including the performance of IIoTMS classification model, system usability, and user satisfaction. These aspects represent important indicators of the system, while it will be compared later to another similar system in the next section.

### 5.1. Performance of IIoTMS

The performance of IIoTMS classification model will greatly affect the marketing of IoT products in the marketplace. As any RS can be evaluated using four classes of metrics: (1) Predictive accuracy. (2) Classification accuracy. (3) Rank accuracy. (4) Non-accuracy [52,53]. 

Classification accuracy was chosen to validate the IIoTMS prediction model because it is the most appropriate metric in this type of application. In addition, it can be used to evaluate the successful decision-making ability of recommendation algorithms. This metric focuses on the amount of correct and incorrect classifications using a confusion matrix. It is a matrix to accumulate the numbers of true/false positive/negative recommendations regarding users’ preferences, as shown in Table 1. 

The *precision* or true *positive accuracy* is calculated as a ratio of recommended items relative to the total number of items recommended. It represents the probability of the recommended product matching to user’s preferences, as follows [52]:

(4) $$\mathrm{precision}=\frac{\mathrm{tp}}{\mathrm{tp}\;+\;\mathrm{fp}}$$

In the same vein, the *true positive rate* (*recall*) is calculated as the ratio of the recommended relevant items to the total number of related items. It represents is the probability of recommending the relevant item, as follows [52]:

(5) $$\mathrm{recall}=\frac{\mathrm{tp}}{\mathrm{tp}\;+\;\mathrm{fn}}$$

The relationship between precision and recall is inverse; increasing the size of the set of recommendations will increase recall but decrease accuracy. As a result, both metrics can be combined into another scale called F1. This scale is used as the standard harmonic mean of precision and recall, which is calculated as [52]:

(6) $$F1=\frac{2\;\left(\mathrm{precision}*\;\mathrm{recall}\right)}{\mathrm{precision}\;+\;\mathrm{recal}}$$

**Figure 12:**
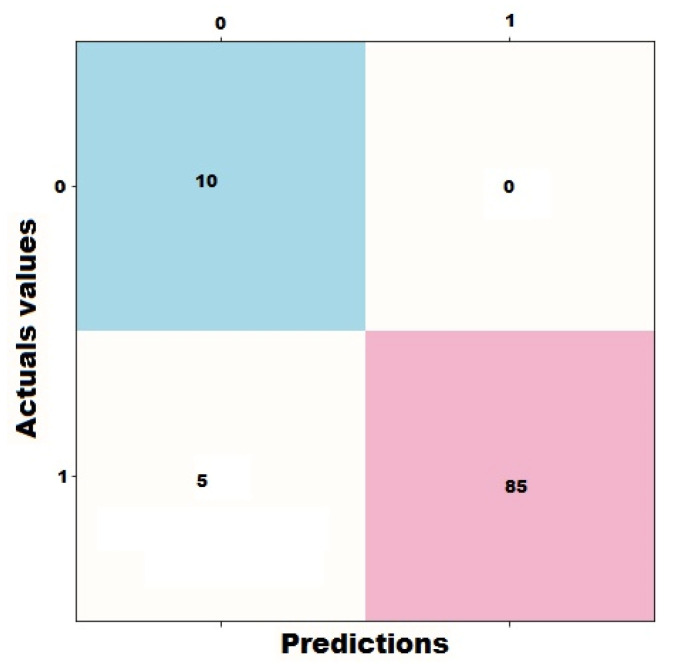
Predictions versus actual values on test data using the confusion matrix.

The IIoTMS classification model was tested using test data containing information about 100 wearable devices. These products are divided as follows: 15 products are rated as 5 stars, 25 products are rated as 4 stars, 27 products are rated as 3 stars, 13 products are rated as 2 stars, and 20 products are rated as 1 stars. We analyzed the resulting recommendations using a confusion matrix. Based on the initial results, predictions versus actual values can be summarized using a confusion matrix, as shown in Figure 12. 

IIoTMS shows good results in its performance which is reflected in a number of relevant metrics, as shown in Table 2. 

We further calculated the accuracy of IIoTMS. It is a measure expressing the ratio of true positives and true negatives to all positive and negative values, as shown in Table 3. The accuracy is an important indicator of how often the machine learning model can correctly predict.

There are many data mining methods and techniques that can be applied to design RS, especially in terms of classification. For instance, Nearest Neighbors, Decision Trees, Ruled-based Classifiers, Bayesian Classifiers, Artificial Neural Networks, Support Vector Machines, and Association Rule Mining. As a result, choosing the right classifier for a specific recommendation task still requires a lot of exploration [24,45,46,48]. It also should be noted that it is necessary to start with the simplest ML approach during the experiments. Complexity is only entered if performance gains are guaranteed because it requires a balance between several dimensions [54].

### 5.2. System Usability

To examine IIOTMS usability, the System Usability Scale (SUS) was used to study the user community. SUS is a focused questionnaire that was created by John Brooke in 1986, which is used to assess various types of computer systems such as software, mobile devices, websites, and applications. It consists of 10 questionnaire items with five responses for the respondents (from strongly agree to strongly disagree) [55]. 

**Figure 13:**
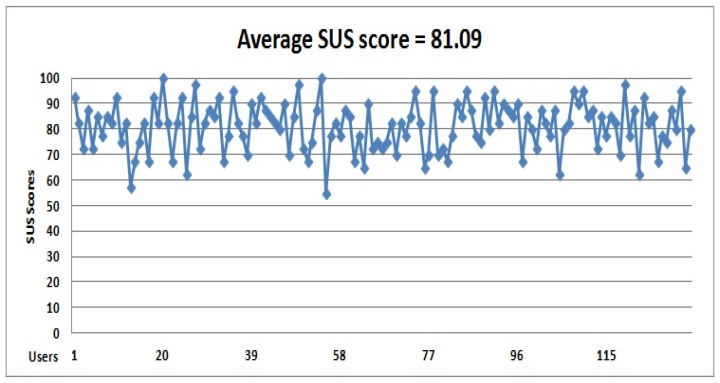
The SUS score of the IIoTMS.

By looking at the purchase records, the number of customers in Rehab can reach more than 2500 customers. It is a large community that requires a random sample of at least 100 participants. Consequently, a sample of 150 users was surveyed, of which 133 participants responded within a specified two-day period, as shown in Figure 13. 

Based on the scale interpretations, the average SUS score is **68**, IIoTMS has a higher usability score (**81.09 = excellent**). Some important indicators are also extracted from the feedback, confirming the positive impression of the application. For example, **90.9%** of the respondents want to use IIoTMS more frequently. A total **85.7%** felt very confident using IIOTMS.

### 5.3. User Satisfaction

**Figure 14:**
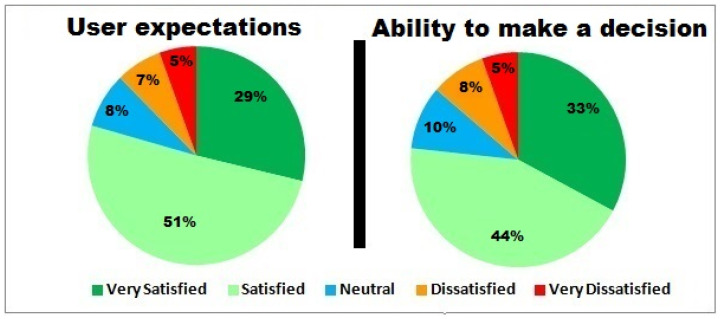
Users’ expectations and their ability to make the right decisions.

**Figure 15:**
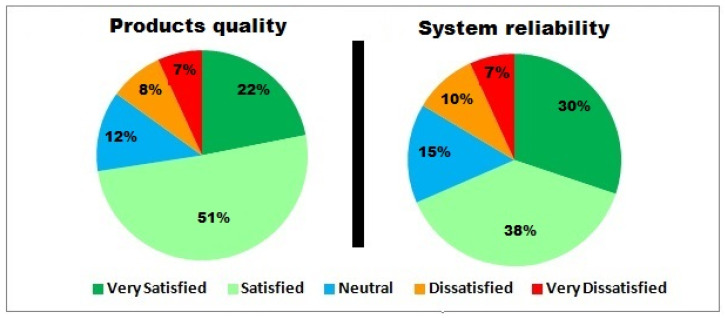
Recommended product quality, and IIoTMS reliability.

The study has also focused on estimating IIoTMS user satisfaction using the Customer Satisfaction Score (CSAT), as shown in Figure 14 and Figure 15. CSAT is a scale that covers aspects related to user satisfaction using simple questions with a binary response. This includes users’ expectations about IIoTMS, decision-making ability, recommended product quality, and reliability.

These results give indications about the system’s ability to meet consumer requirements regarding product quality.

## 6. Discussion

No doubt that recommending products in IoT is crucial to facilitating the popularity of this field. However, leading a new consumer to find relevant products that meet their needs with quality in mind is a very complex process. It mainly depends on meeting their explicit and implicit requirements.

Much effort has been made in this direction, but it seems that this is not enough. This study sought to do more by suggesting a consumer-oriented approach based on an effective recommendation technique. It takes into account the experiences of past consumer experts as key players in defining/assessing the quality characteristics of IoT products. In addition, it has relied on hybrid filtering techniques to make different comparisons between data items (products and users). This technique allows combining CB and CF techniques, to obtain accurate predictions as well as to make some heuristics.

Most of the hybrid RS apps can be implemented using the same machine learning algorithms but the difference is in the accuracy of recommendations. It appears that the IIoTMS is very conservative when it comes to predicting low-rated products. In addition, it has a very high accuracy of up to **95%**. Moreover, **80%** of users seem to feel that IIoTMS meets their expectations, while **77%** are able to make the right decision before purchasing any IoT product. Furthermore, **83%** of users are satisfied with the quality of products recommended by IIoTMS. A total **68%** of users felt IIoTMS is reliable.

The results show that IIoTMS as an IoT recommender is more effective than others. This happens because the applied approach allows the integration of many different elements, such as concepts, data, methods, and technologies, to provide more useful recommendations. Indeed, there are many application scenarios for recommender systems in various fields. The use of these systems in the areas of smart shopping or smart marketing is the most interesting scenario. It draws the attention of many researchers; several architectures are proposed to connect IoT devices with each other, including the Social Internet of Things (SIoT) and the Multi-Internet of Things (MIoT). Accordingly, the proposed approach is worthy enough to be applied in complex architectures such as those presented before as in [27,56]. It provides a general framework that includes all the actors required to make useful recommendations for IoT, including vendors or suppliers, consumers and quality experts. It also takes into account potential interactions between smart devices in a distributed environment.

For more credibility, the discussion will also focus on comparing IIoTMS with another similar IoT recommendation system. In [28,29], the authors proposed IoTSRS to recommend users with IoT services based on the object they own. This recommender uses a graph-based model that analyzes various entities and their relationships, including objects, services, and users. Although the graph-based approach has shown promising results in various fields, the current IoT service recommendation model is very primitive. For instance, IoTSRS is not able to deal with the sensor characteristics, which affect their ability to provide useful recommendations (i.e., sensor location, and mobility). IoTSRS has also not implemented a clear methodology to meet the needs of the user, while IIoTMS considers the application of a consumer-oriented approach to meet their needs. To substantiate this claim, results regarding the accuracy of the recommendations, system usability, and user satisfaction can be referred to in Section 5.

There are three principles that were followed during the development of IIoTMS that helped achieve these good results. It can be summarized as follows: (1) Focus on the tasks that will be performed by the users. (2) Follow clear methodologies in designing the system and assessing product quality. (3) Adopting machine learning approaches in the recommendation and a number of smart technologies. Accordingly, we can argue that the proposed IoTSRS is less effective than IIoTMS.

The results not only demonstrate the validity of assumptions, but can also be understood as conclusive evidence that the hybrid approach works reasonably well for recommending IoT products. There are many interesting questions that can be drawn for future research. Future researchers can extend the research to address typical limitations in recommender systems, such as cold start problems, sparsity, scalability, overspecialization, grey sheep problem, etc. Some researchers can continue to explore the quality factors of the IoT in an advanced way. The biggest concern is the lack of a reference model for IoT quality.

## 7. Conclusions

In this paper, we propose a new approach to assessing IoT products and recommending high-quality products to new consumers. We have seen that new consumers of IoT face different challenges to choose smart products that best suit their needs. We have also noticed that IoT markets are filled with many poor-quality products, especially in terms of device quality. Therefore, we have presented a consumer-oriented approach to help those potential consumers. The proposed approach is based on techniques of recommendation systems in which different elements are combined together to provide useful recommendations. We focused on applying hybrid techniques, as they allow data mining and avoid shallowness. Consumer requirements are thus met using several levels of recommendations, including filtering items based on initial search conditions, filtering items based on consumer’s interests, filtering items based on consumer’s constraints, and utility calculations for the recommended items. Afterward, we made our experiments to implement this approach at the Rehab marketplace. The applications scenario is an Intelligent IoT Marketing System (IIoTMS). IIoTMS is a distributed system consisting of several components in which a number of users interact according to predefined roles and permissions. System tests demonstrated the applicability of the proposed approach. It provides accurate recommendations, ease of use, and user satisfaction.

The study represents a real contribution in terms of combining recommendation and IoT technologies. The proposed approach could work in many areas to serve consumers, particularly in connection with the outbreak of the COVID-19 pandemic, as all computer-based recommendation systems are becoming increasingly common and necessary. It can also be implemented using complex architectures such as MIoT and SIoT.

## Figures and Tables

**Table 1:** The elements of confusion matrix.

Title 1	Relevant	Irrelevant	Total
Recommended	tp	fp	tp + fp
Not Recommended	fn	tn	fn + tn
Total	tp + fn	fp + tn	N

tp: true positive, fp: false positive, fn: false negative, tn: true negative.

**Table 2:** The performance of IIoTMS classifier.

Measure	Value
Precision	1.0000
Negative Predictive Value	0.9444
False Positive Rate	0.0000
False Discovery Rate	0.0000
False Negative Rate	0.3333
F1 Score	0.8000

**Table 3:** The accuracy of the IIoTMS.

Measure	Value
Accuracy	0.9500

ACC = (TP + TN)/(P + N).

## Data Availability

We did not report any data.
